# Multi-*b* value diffusion-weighted magnetic resonance imaging and intravoxel incoherent motion modeling: Differentiation of aggressive lymphoma lesions on initial treatment and activity assessment after chemotherapy

**DOI:** 10.1097/MD.0000000000014459

**Published:** 2019-02-08

**Authors:** Cheng-cheng Liao, Yun-ying Qin, Qi Tang, Xiao-hong Tan, Qing Ke, Yan Rong, Jian-Hong Zhong, Le-qun Li, Hong Cen

**Affiliations:** a Department of Chemotherapy; b Department of Radiology; c Department of Hepatobiliary Surgery, Affiliated Tumor Hospital of Guangxi Medical University; d Guangxi Liver Cancer Diagnosis and Treatment Engineering and Technology Research Center, Nanning, P.R. China.

**Keywords:** cell diffusion, diffusion weighted imaging, intravoxel incoherent motion, lymphoma, magnetic resonance imaging

## Abstract

The diagnostic efficiency of diffusion-weighted magnetic resonance imaging with different *b*-values and application of an intravoxel incoherent motion (IVIM) model for differentiating disease states of lymphoma was investigated.

Thirty-six patients at initial diagnosis and 69 after chemotherapy underwent diffusion-weighted magnetic resonance imaging (DW-MRI) with multiple *b*-values. Analysis parameters included the apparent diffusion coefficient (ADC) for each *b*-value. Standard ADC, *D*, *D*∗, and *f* were calculated using an IVIM model.

For patients at initial diagnosis, compared with aggressive lymphomas, the benign lymph nodes exhibited higher mean ADC (2.34 vs 0.66 × 10^−3^ mm^2^/s, *P* < .01) for *b* = 200 s/mm^2^. The AUC, sensitivity, specificity, and the cutoff value were 0.992, 96%, 100%, and 1.09 ×10^−3^ mm^2^/s, respectively. For patients who had finished chemotherapy, the *f*-values of IVIM for those with partial remission (PR) were higher than those of complete remission (CR) (56.22 vs 21.81%, *P* < .01). The AUC, sensitivity, specificity, and the cutoff value were 0.937, 94%, 82%, 42.10%, respectively.

For *b* = 200 s/mm^2^, ADC values are most helpful for characterizing benign lymph nodes and malignant lymphomas. The *f*-value of the IVIM is most valuable in the identification of residual lesions of lymphomas after chemotherapy.

## 1 Introduction

The staging and therapeutic evaluation of lymphoma can guide the formulation of treatment protocols and change of the therapeutic regimen when appropriate. Accurate staging and therapeutic evaluation can maximize efficiency and minimize treatment-related adverse events. Imaging tests, including enhanced computed tomography (CT) and B ultrasound scans are the most common approaches for evaluation of lymph nodes but lack diagnostic efficiency.^[1]^ Currently, fludeoxyglucose (^18^FDG) positron emission tomography (PET/CT)^[2]^ is the recommended method for initial staging of lymphoma by many international guidelines recommended for detection of remission of diffuse large B-cell lymphoma and Hodgkin lymphoma. While PET/CT is a valuable tool, it exposes patients to ionizing radiation. Further, the diagnostic sensitivity and specificity and the histological resolution are low when dealing with lymphomas.^[3]^ Previously, magnetic resonance imaging (MRI) was mainly utilized for regional examinations, but imaging techniques have advanced and now include respiratory gaiting and imaging of the abdomen. Intravoxel incoherent motion (IVIM) DW-MRI can separately determine the diffusion of water molecules and microcirculatory perfusion. Conventionally, the apparent diffusion coefficient (ADC) value determined with DWI can predict the efficiency of chemotherapy.^[4]^ However, the specificity of an ADC value is unsatisfactory as it is affected by perfusion and diffusion. This investigation studied newly diagnosed patients and patients who achieved complete remission (CR) or partial remission (PR) after chemotherapy via pathological or follow-up studies. Scans were obtained with various *b*-values and the IVIM model were compared. This study aimed to provide a new imaging alternative without ionizing radiation for the staging, diagnosis, and prognostic prediction of malignant lymphomas.

## 2 Materials and methods

This research met the Declaration of Helsinki and was approved by the Ethics Committee of the Affiliated Tumor Hospital, Guangxi Medical University. All patients signed an informed consent. The MRI sequence parameters were investigated under the prospective clinical trial NCT02733887.

Patients were divided into a newly diagnosed group and a post-chemotherapy group (group C). The newly diagnosed group was further divided into a group with benign lymph nodes (group A, control group) and a group with aggressive lymphoma (group B). The post-chemotherapy group was also further divided into those who achieved complete remission (group C1) and those who did not (group C2). All patients were aged ≥18 years, and recruitment started in December 2014.

### 2.1 Patient and public involvement

MRI is a non-ionizing radiation test designed to assess the efficacy of staging of lymphoma patients with new scan parameters. The doctors of our chemotherapy department discuss the feasibility and significance of this project. As this study shows the advantage, we will conduct the next controlled trial. All patients came from our hospital clinic. In the newly diagnosed group, we recruited highly suspected lymphomas (excluding solid tumors) and those suspected of having benign lymphadenopathy. And patients are willing to lymph node biopsy. In the chemotherapy end group, we primarily scanned patients with residual disease after chemotherapy. Two patient representatives are members of the Li Qiang and Liu Zhi Hui and have been provided with research training and support to ensure they understand their role in the research project. The results of the study will be released in phases to study participants by project management members.

### 2.2 Group characteristics

(1) Newly diagnosed group: clinically suspected of lymphoma (excluding those with solid tumors and lymphatic metastasis as much as possible) and willing to undergo MRI as well as total resection of lymph nodes for biopsy. Patients were eliminated from the group if the pathological tests of lymph nodes indicated non-aggressive lymphoma or non-benign lymph nodes.
(2) Post-chemotherapy group:

a. Imaging tests (CT or MRI) after chemotherapy of those with aggressive lymphomas suggested that the major targeted lesions were all measurable lymph nodes (excluding extra-nodal lesions) with a longest transverse diameter (LDi) >1 cm.
b. Patients who achieved stable disease or progressive disease (PD) as evaluated via enhanced CT or MRI after their last dose of chemotherapy were excluded.
c. Patients who could be followed regularly (every 2 months) with CT or MRI scans (no <18 months) of the residual lesions for evaluation and subsequent follow-up or who went through residual lesion resection for biopsy after MRI (1 month after chemotherapy).

Diagnosis and histological classification of all lymphoma patients was based on the WHO classification of tumors of hematopoietic and lymphoid tissues (2008).^[5]^

### 2.3 Residual lesion evaluation and follow-up

As the post-chemotherapy group, the major endpoint was duration of response (DOR), which was defined as the time from the moment CR or PR was achieved to when PD was detected or death occurred due to any reason, based on Cheson et al.^[6]^ If the residual lesions could not be resected for biopsy, patients from the post-chemotherapy group were followed according to:

1. Therapeutic efficacy assessment (via MRI/CT) 1 month after chemotherapy (patients who achieved PD were excluded). Cheson et al^[6,7]^ criteria was used for determination of efficacy.
2. After chemotherapy, MRI or enhanced CT scans were performed at the first month and then every 2 months. Patients were immediately sent for re-examination in the case of relapse or relevant clinical symptoms.
3. Absence of any anti-neoplastic therapy before relapse.
4. All patients with relapse were confirmed by pathological or imaging (the recurrence of imaging is defined as: according to the Cheson et al^[6,7]^ criteria, the previous target lesion reached PD).
5. The major endpoint of follow-up was the duration of response (DOR) or a follow-up period >18 months.
6. Patients were assigned to group C2 (not achieving CR) if endpoint events were observed (such as PD). Follow-up was terminated when the follow-up period of the last included patient reached 18 months. Those who did not show PD during the follow-up were assigned to group C1 (achieving CR).
7. Data of patients who were lost to follow-up were censored.

### 2.4 MRI schemes and parameters

A 3.0-T MRI scanner (GE discovery 750W, Chicago, IL) was used in this research. Scans of the nasopharynx and neck were completed with a head and neck coil under free breathing. Scans of the chest, abdomen, and pelvis were completed with an abdominal coil and respiratory gating was employed. Conventional MRI and multi-*b*-value DW images were collected before treatment, at mid-stage evaluation, and when conducting therapeutic efficiency assessment after chemotherapy. The value of *b* was set at 0, 30, 50, 80, 100, 150, 200, 400, 600, 800 s/mm^2^ and varied throughout a single imaging session. Imaging parameters are shown in Table 1.

**Table 1:**
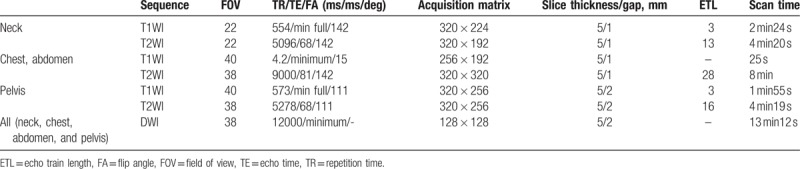
Magnetic resonance image acquisition parameters.

The equation of the biexponential model used in this research is: 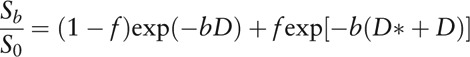


Where *b* is the diffusion-sensitive gradient factor, *S*_0_ is the signal intensity of DWI when *b* was 0 s/mm^2^, and *S*_*b*_ is the signal intensity of DWI when *b* was >0 s/mm^2^. *D* is the true diffusion coefficient and it reflects the pure diffusion of molecules. *f* is the perfusion fraction and it reflects the proportion of false diffusion, which forms by microcirculatory perfusion in IVIM. *D*∗ is the false diffusion coefficient and it reflects the perfusion-related diffusion coefficient. The true diffusion coefficient could be calculated after multi-*b*-value diffusion signals were linearly fitted. All the coefficients were fitted with a non-linear least square method and perfusion-related diffusion coefficients were calculated.

The GE Medical Systems Functool 9.4.05 (GE Healthcare, Waukesha, WI) was used to reconstruct the ADC diagram. The maximum cross-sectional area of the lesion for each region of interest (ROI) was selected and the ADC was obtained, using the T2-weighted images for morphological reference. The monoexponential model was used to measure the above-mentioned *b* values for the ADC values and sADC values. The *D*, *D*∗, *f*, values were measured using a bi-exponential model.

### 2.5 Statistical analysis

Patient demographics and baseline characteristics of the group A, B, C1, and C2 were recorded. Median follow-up of Group C1 was 22 months (18–24 months). Median follow-up of Group C2 was 3 months (1–6 months). Patient characteristics were analyzed using a Mann–Whitney *U* test. One-way ANOVA was adopted to analyze differences in ADC values at different scan sites and the high-risk factors including age, sex, Eastern Cooperative Oncology Group (ECOG) score, lactate dehydrogenase (LDH) levels, LDi of lymph node, ADC, *D*, *D*∗, and *f*-values. A multi-variate logistic regression model was adopted to evaluate the important variables screened out by the ANOVA.

Benign and malignant pathological results were set as dependent variables in the newly diagnosed group. In the post-chemotherapy group, any relapses occurring during follow-up and benign and malignant pathological results were set as dependent variables (assigned as 0 and 1 respectively in logistic regression). The null variables in the one-way ANOVA were eliminated and the remaining variables were screened again. A maximum likelihood estimation was employed to establish a logistic regression model.

R package was utilized for statistical analysis (R Core Team (2016) Vienna, Austria). All *P* values were examined with double precision and *α* = 0.05.

## 3 Results

### 3.1 Subjects

**Figure 1:**
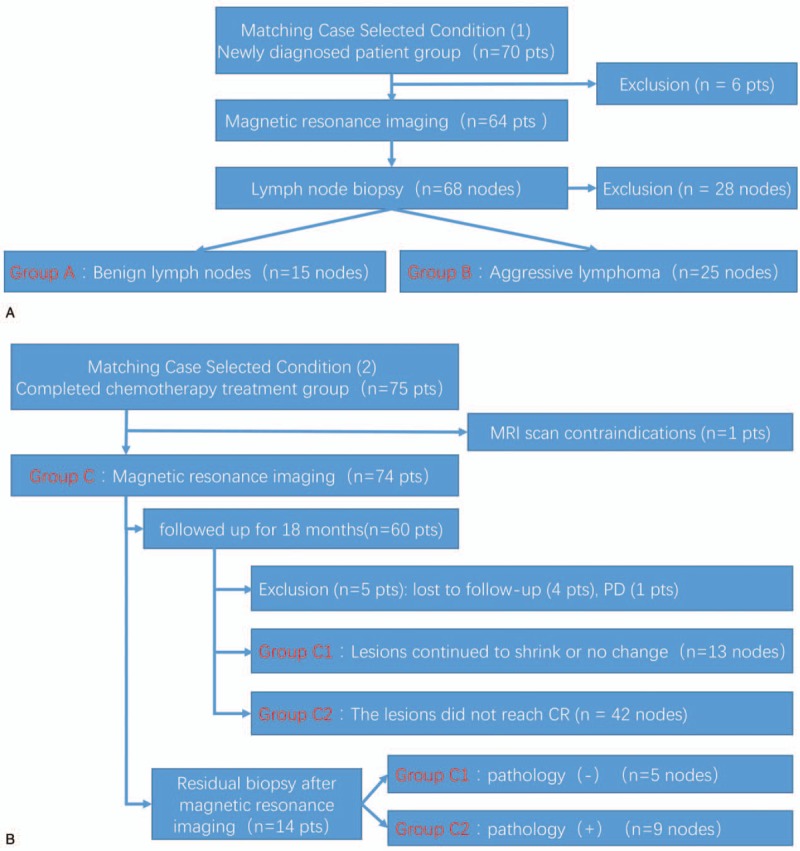
Flow chart of case screening for the newly diagnosed group (A) and the post-chemotherapy group (B).

This study included a total of 145 patients. After screening based on the inclusion criteria (Fig. 1), 36 patients were included into the newly diagnosed group (15 benign lymph nodes and 25 aggressive lymph nodes) and 69 patients were included into post-chemotherapy group for further analysis (Tables 2 and 3).

**Table 2:**
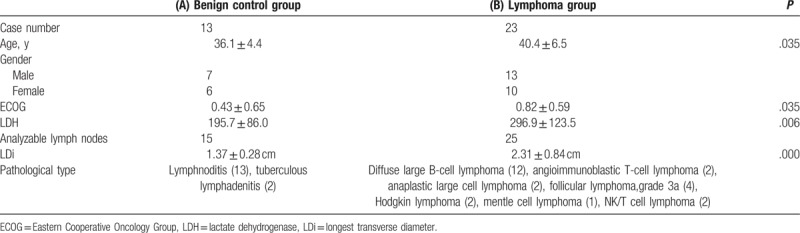
Patient characteristics for the newly diagnosed group.

**Table 3:**
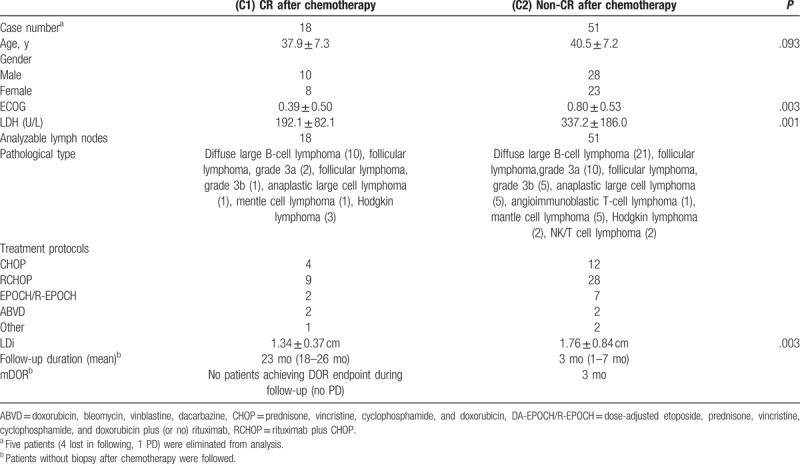
Patient characteristics for the post-chemotherapy group.

### 3.2 Quantitative analysis

#### 3.2.1 Size of lymph nodes

In the newly diagnosed group, the LDi of benign lymph nodes in the control group was significantly shorter than that of the malignant lymphoma group (Table 2, *P* < .001). The area under the curve (AUC) was 0.828. The optimal threshold for distinguishing benign from malignant lymph nodes was a long diameter ≥2.08 cm. In this study the accuracy rate was 81%, with a sensitivity of 100%, and specificity of 68%.

In the post-chemotherapy group, the LDi of CR lymph nodes was significantly shorter than that of the PR group (Table 3, *P* = .003). The AUC for this group was 0.669. The optimal threshold for distinguishing CR from PR lymph nodes is an LDi ≥1.23 cm. In this analysis, the accuracy rate was 72%, with a sensitivity of 61%, and a specificity of 76%.

Differences in ADC values at different scanning positions ADC values (*b* values = 0–800 s/mm^2^) were divided into 4 parts: neck (3.04 ± 2.61) × 10^−3^ mm^2^/s, chest (2.95 ± 2.46) × 10^−3^ mm^2^/s, abdomen (2.95 ± 2.37) × 10^−3^ mm^2^/s, pelvis (2.84 ± 2.17) × 10^−3^ mm^2^/s in the newly diagnosed group and the post-chemotherapy group according to the magnetic resonance scanning site. There was no significant difference in ADC values among the 4 groups (*P* > .05).

#### 3.2.2 ADC values of lymph nodes with different *b*-values

**Figure 2:**
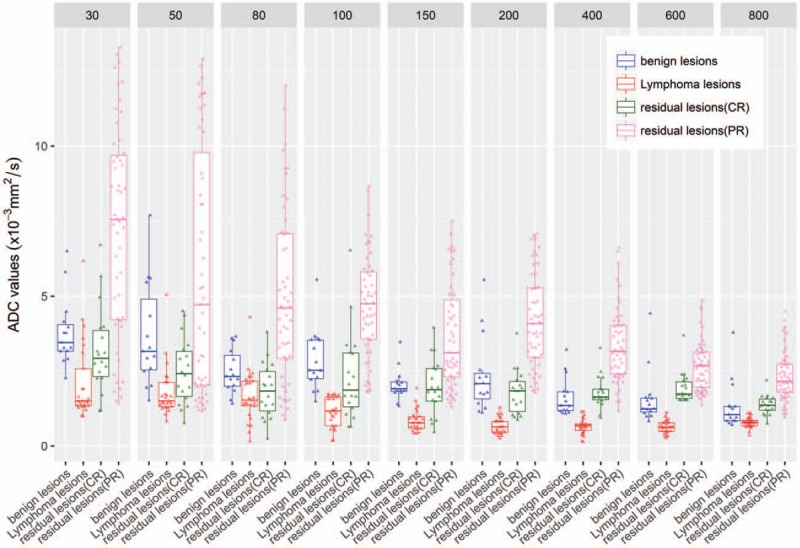
Discrimination of the type of lymph nodes with multi-*b*-values.

In the newly diagnosed group, for all *b*-values, the mean ADC value of benign lymph nodes in the control group was significantly higher compared with the malignant lymphoma group (*P* < .01). The maximum diagnostic efficiency was observed when *b* = 200 s/mm^2^ (Table 4). The optimal threshold for distinguishing benign from malignant lymph nodes was observed for a mean ADC (mADC) = 1.09 × 10^−3^ mm^2^/s (Table 4). In the post-chemotherapy group, for all *b*-values, the average ADC value of the lymph nodes of patients who achieved CR was significantly lower than that of those who achieved PR (*P* < .01). The maximum diagnostic efficiency was observed when *b* = 200 s/mm^2^. The optimal threshold for distinguishing PR of lymph nodes and CR of lymph nodes after chemotherapy was observed for a mADC = 2.65 × 10^−3^ mm^2^/s (Table 5). The boxplot in Fig. 2 shows the difference in the average ADC value between the benign lymph nodes and malignant lymphoma in the newly diagnosed group, and between the lymph nodes with PR and those with CR after chemotherapy.

**Table 4:**
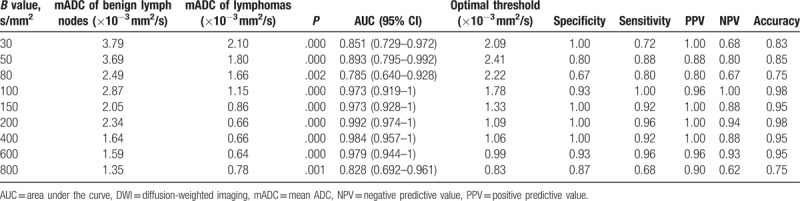
Distinguishing between benign lymph nodes and malignant lymphomas using multi-*b*-value DWI.

**Table 5:**
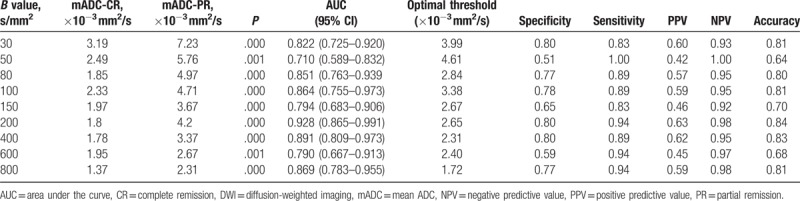
Distinguishing between lymph nodes with PR and lymph nodes with CR after chemotherapy using multi-*b*-value DWI.

#### 3.2.3 Quantitative analysis of lymph nodes under IVIM model

In the newly diagnosed group, the average Standard ADC (sADC) value of the benign lymph nodes in the control group was significantly higher than that of the malignant lymphoma group (*P* < .01). The AUC for this group was 0.987 (95% CI, 0.962–1). The optimal threshold for distinguishing benign and malignant lymphomas was observed for a sADC = 0.49 × 10^−3^ mm^2^/s (Table 6).

**Table 6:**
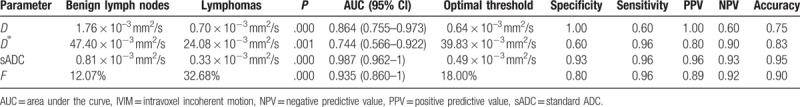
Distinguishing between benign lymph nodes and malignant lymphomas using IVIM model.

**Figure 3:**
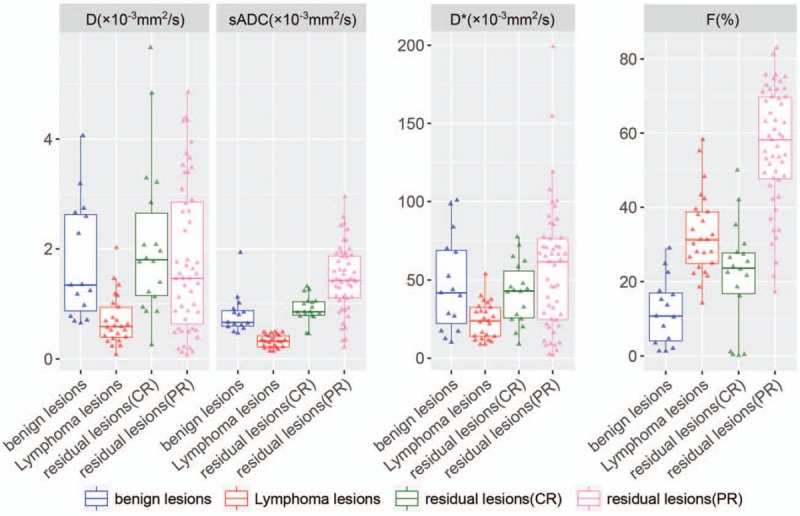
Discrimination of the type of lymph nodes with IVIM. IVIM = intravoxel incoherent motion.

**Figure 4:**
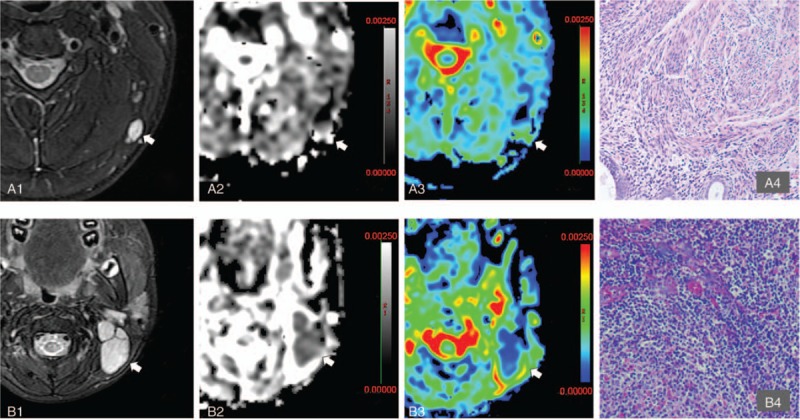
Example MRI images of patients in the newly diagnosed group. A1–4 30 years old, man, left cervical chronic lymphadenitis. A1 is T2WI, the left cervical lymph node is high signal; A2 is the ADC map (*b* = 200 s/mm^2^), the arrow shows that the lymph node was middle-high mixed signal, ADC = 2.12 × 10^−3^ mm^2^/s; A3 is sADC map. The arrow showed the lymph node was a moderate signal, sADC = 1.25 × 10^−3^mm^2^/s; A4 for the pathological picture (100×). B1–4 women, 42 years old, follicular lymphoma grade 3a. B1 is T2WI, the left cervical lymph node is high signal; B2 is the ADC map (*b* = 200 s/mm^2^). The arrow showed that the lymph nodes were low signal, ADC = 0.55 × 10^−3^mm^2^/s; B3 is the sADC map, arrows show lymph nodes low signal, sADC = 0.23 × 10^−3^mm^2^/s; B4 is the pathological picture (100×). ADC = apparent diffusion coefficient, MRI = magnetic resonance imaging, sADC = standard apparent diffusion coefficient; T2WI = T2 weighted image (transverse relaxation time weighted image).

**Figure 5:**
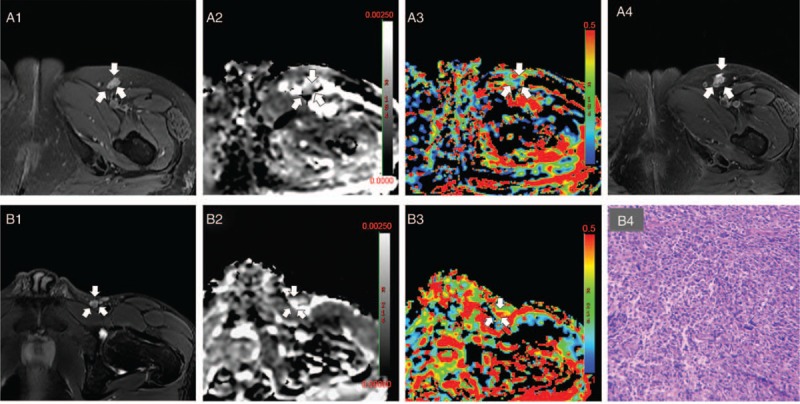
Example MRI images of patients in the post-chemotherapy group. A1–4 women, 45 years old, diffuse large B-cell lymphoma. A1 for the T2WI, the left inguinal lymph node was high signal; A2 is ADC map (*b* = 200 s/mm^2^). The arrow showed lymph node was low-middle mixed signal, ADC = 1.08 × 10^−3^ mm^2^/s; A3 is the sADC map, arrow showed lymph nodes medium signal, *f* = 30.2%; A4 is the 18th month of MRI T2 follow-up picture, no significant changes in lesions, indicating complete remission of lesions. B1–4, man, 42 years old, diffuse large B cell lymphoma. B1 is the T2WI, left inguinal lymph node was high signal; B2 is the ADC map (*b* = 200 s/mm^2^), the arrow showed a high signal lymph nodes, ADC = 2.61 × 10^−3^ mm^2^/s; B3 is the *f* map, the arrow showed high signal lymph nodes, *f* = 49.3%; B4 for lymph node biopsy pathology (100×). ADC = apparent diffusion coefficient, MRI = magnetic resonance imaging, sADC = standard apparent diffusion coefficient.

In the post-chemotherapy group, the average *f*-value of the lymph nodes of patients who achieved CR after chemotherapy was significantly lower than that of those who achieved PR (*P* < .01). The maximum diagnostic efficiency was obtained when the *f*-value was used for measurements. The AUC for this group was 0.937 (Table 7). The optimal threshold for distinguishing PR of lymph nodes and CR of lymph nodes after chemotherapy was observed for an *f* = 42.1% (Table 7). The boxplot in Fig. 3 shows the differences in analysis parameters between benign lymph nodes and malignant lymphomas in the newly diagnosed group, and between lymph nodes of PR and lymph nodes of CR after chemotherapy. Representative images are shown in Figs. 4 and 5.

**Table 7:**
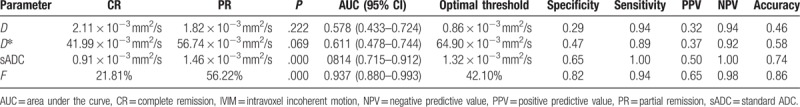
Distinguishing between lymph nodes with PR and lymph nodes with CR after chemotherapy using IVIM model.

### 3.3 Evaluation of consistency among observers

The two radiograph reviewers that only knows some clinical information except for follow-up and pathological findings, each with 15 years of experience demonstrated a mean percentile difference of 0.8% (95% confidence interval [CI], 0.6–2.1%) when measuring the size of the lymph nodes (intraclass correlation coefficient [ICC] = 0.94; 95% CI 0.91–0.96). They also showed a mean percentile difference of 1.2% (95% CI, 0.6–3.1%) when measuring ADC values ICC = 0.92; 95% CI 0.91 to 0.93. These results indicated that the 2 radiograph reviewers were highly consistent.

### 3.4 Regression forecasting model

**Figure 6:**
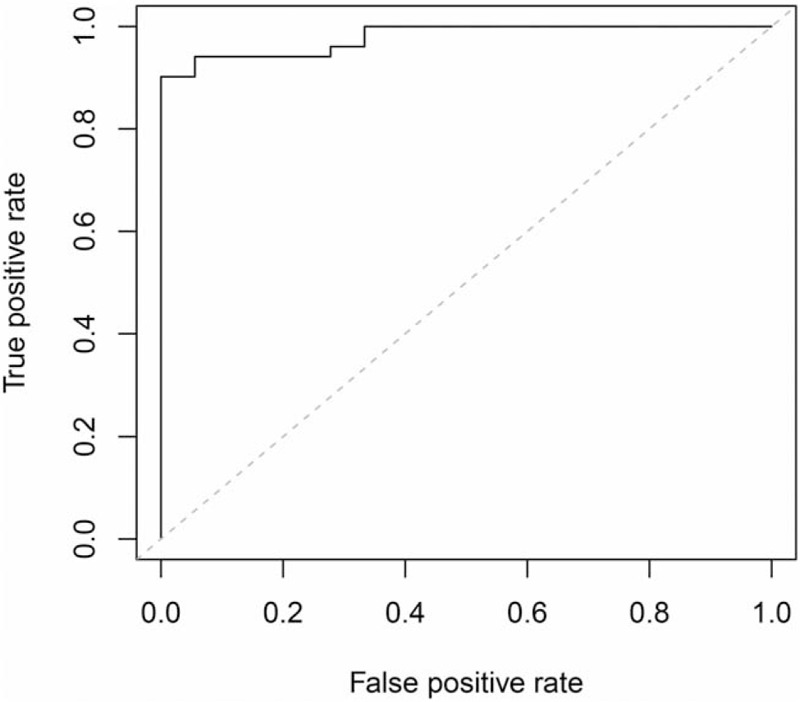
Logistic regression combined with multivariate diagnostic ROC curve. ROC = receiver operating characteristic curve.

A logistic regression model could not be established for the newly diagnosed group. For the post-chemotherapy group, the logistic regression model was as follows: 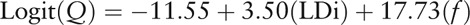


A *Q* < 0.5 was considered as an achievement of CR, whereas a *Q* > 0.5 was considered as a failure to achieve CR. The accuracy rate was 93% (Fig. 6).

## 4 Discussion

The precise staging and efficacy evaluation of lymphoma is particularly important for the use of appropriate treatment plans. PET/CT can be anatomical and metabolic fusion imaging, as invasive in lymphoma staging and efficacy evaluation of the preferred means. But PET/CT also has its flaws, such as PET/CT itself exists ionizing radiation, pregnancy, lactating women, and contrast agent allergies are not applicable. MRI in the past was considered to have high frequency of motion artifact is not suitable for full body scan. However, with the innovation of magnetic resonance technology, high frequency of motion artifact can be significantly improved by using the corresponding coil and breathing gating technology. Diffusion-weighted imaging (DWI) is a noninvasive technique to reflect the diffusion characteristics of water molecules in living tissue. The functional imaging of DWI with multiple *b* values can obtain richer diagnostic information. Making magnetic resonance for lymphoma staging and efficacy evaluation possible. Whole-body MRI for the study of lymphoma at present uses a “PET-like” imaging sequence,^[8,9]^ and has been used for initial staging and therapeutic assessment for lymphoma. Currently, therapeutic evaluation and staging is usually based on the ADC value obtained from a monoexponential model with 2 or 3 *b*-values. However, ADC is affected by both perfusion and diffusion. When the *b*-value is relatively low, the impact of blood perfusion denotes differences between the proliferation of water molecules in benign and malignant tumors, decreasing the diagnostic performance.^[10]^ If the *b*-value is increased to eliminate this effect, the resulting signal to noise ratio can also reduce the diagnostic accuracy.^[11]^ Moreover, such studies have almost no pathologic and/or long-term follow-up validation of MRI functional imaging features for the initial staging and efficacy evaluation of lymphoma.

Studies have demonstrated that the mean ADC value of malignant lymphomas was lower than that of benign lymph nodes.^[12,13]^ Wu et al^[14]^ conducted a preliminary evaluation on diffuse large B-cell lymphomas and follicular lymphomas with 2 *b*-values (*b* = 0 and 800 s/mm^2^) and obtained a mean ADC value of (0.70 ± 0.16) × 10^−3^ mm^2^/s and (0.76 ± 0.12) × 10^−3^ mm^2^/s, respectively, which is consistent with our findings.

When distinguishing between benign lymph nodes and malignant lymphomas in the newly diagnosed group, a *P* < .05 always accompanied a low *b*-value, such as <1 × 10^−3^ mm^2^/s. A possible mechanism for this difference between benign lymph nodes and malignant lymphomas is due to extracellular diffusion of water molecules. Lyng et al^[15]^ found that tumors with a high cell density were characterized by translucent cell membranes with lower permeability and higher intracellular water content. Smaller extracellular space and lower cell density limits the diffusion of free water molecules, which positively correlates to the ADC values observed with MRI. Cell,^[15,16]^ animal experiments,^[17]^ and clinical studies^[18,19]^ have shown that tumor cell density, tumor cell dysplasia, pathologic type, nucleus/cytoplasmic ratio, and mesenchymal components are also factors affecting ADC values. In the newly diagnosed group and post-chemotherapy group, we can see from the statistical chart that with the increasing of *b* value, the sensitivity and specificity increased first and then decreased. According to the principle of diffusion weighted imaging, when the *b* value is low, the diffusion of water molecules is not sensitive, and the attenuation of tissue signals is much influenced by blood perfusion and microcirculation. High *b* value can reduce the influence of T2 penetration effect, and ensure that the ADC value measurement is more accurate. High *b* value is more likely to producing magnetic sensitive artifacts, image geometric deforming more serious, and image quality declining. And high *b* value means that a long TE, the signal-to-noise ratio will significantly decline for the lymph nodes which have shorter T2 values. Therefore, *b* value should be chosen moderately, to take care of both the DWI image quality and sensitivity to the lesion display. Our research suggested that, when *b* = 200 s/mm^2^, the signal-to-noise ratio and the detection of diffuse signal in MRI achieved the best balance.

Auser et al^[20]^ studied the IVIM and ADC values of squamous carcinomas of the head and neck, finding that high pre-treatment ADC and *f*-values usually indicated poor therapeutic effect. Barajas et al^[21]^ found that the therapeutic effect on lymphomas in the central nervous system with high ADC values was better than those with low ADC values. When the IVIM double exponential model was applied, the mADC of lymphomas identified at multiple *b*-values was lower than benign lymph nodes in the newly diagnosed group and the sADC of lymphomas was lower than the sADC of benign lymph nodes.

Clinically, it is very important to distinguish between benign lymph nodes and malignant lymphomas between tumor residue (PR) after chemotherapy and CR of tumors after chemotherapy. With CR, residual lesions are often necrotic and comprised of fibrotic tissues. The former is related to disease staging and prognostic prediction, and the latter concerns the need for follow-up treatment. For example, lymphoma would be considered as refractory if CR is not achieved even after 4 to 6 cycles of chemotherapy. For these patients, localized radiotherapy, autologous stem cell transplantation, or clinical trial participation are often then considered. MRI has the potential to assist with these types of clinical decisions. In the post-chemotherapy group of this study, the mADC values across multiple *b*-values of the residual lesions in patients who achieved PR were all lower than that of the residual lesions in those who achieved CR. The IVIM model demonstrated that the *D* value of the residual lesions in patients who achieved PR was lower than that of the residual lesions in those who achieved CR, and that the *f*-value of the residual lesions in patients who achieved PR was higher than that of the residual lesions in those who achieved CR. It was found that the diagnostic efficiency of *f* was optimal comparison metric. Barajas et al^[21]^ found that the therapeutic efficiency of lymphomas in the CNS was better for a group with high ADC values than for low ADC values. Wu et al^[14]^ studied diffuse large B-cell lymphomas with 2 *b*-values (0 and 800 s/mm^2^) and reported the mean ADC value was (0.68 ± 0.18) × 10^–3^ mm^2^/s before chemotherapy and (1.22 ± 0.27) × 10^–3^ mm^2^/s after chemotherapy. In the present study we found that in the post-chemotherapy group, the mean ADC value of patients achieving CR was higher than newly diagnosed lymphoma group (no chemotherapy), however, it was lower than that of those achieving PR. Thus, lower ADC values indicated a longer duration of response. The pathological results of patients achieving CR suggested fibrous tissue/scar tissue remnants. Fibrotic scar tissues may limit the diffusion of free water molecules, causing low ADC values.

All sub-grouping of *b*-values showed statistically significant ADC values. This observation revealed a relatively strong inhibition of chemotherapy on tumor growth. The lesions in the CR group were mainly residual fibrotic scars with low micro-perfusion, leading to an increased *D* with lower *b*-values. Residual lesions in the CR and PR groups after chemotherapy may be due in part to intracellular and extracellular diffusion of water molecules. Since the lesions in the CR group were mainly necrotic and comprised of residual scar tissues, PR group with residual lesions appeared more new blood vessels than the CR group, the *f*-value was lower than that of the group with tumor residues, reflecting the proportion of blood flow. It is affected by tumor microvessel density (MVD). Our study found that *f* value of patients with PR higher than the patients with CR. The reason is related to neovascularization in tumor tissue. *D*∗ values reflect the false spread between tumor cells (microcirculation infusion score), and our study did not show a statistically significant difference. The possible reason is that the difference in true perfusion scores between tumor tissue and lymphoma is not significant. In addition, the proportion of nuclear pulp, the number of cell interstitial components, will also affect the *D*∗ value.

This research utilized a logistic regression model to screen the key indicators in distinguishing between benign lymph nodes and malignant lymphomas, and verify an existing logistic regression model. In the newly diagnosed group, the logistic function did not converge and a regression model could not be established. In the post-chemotherapy group, a *Q* > 0.5 was considered as CR, and the accuracy rate was 93% for an AUC = 0.97. The result of this regression model was superior to PET/CT imaging surveillance of lymphoma.^[22,23]^ However, our research has drawbacks. Firstly, the sample size in this research is relatively small and the number of cases in each subgroup is not equal. There are some impacts on validity of statistical analysis. Although in this exploratory study, meaningful results have been obtained with this sample size and data, it is still necessary to expand the sample for adequate training for the regression model. Secondly, median follow-up duration is relatively short. According to several PET/CT clinical study, Pregno study,^[24]^ in which 88 cases of newly diagnosed diffuse large B-cell lymphoma, the PFS was about 83% when observed 18 months, and 77% when 24 months. In Micallef study,^[25]^ a total of 81 cases of diffuse large B-cell lymphoma, the PFS was about 83% when observed 18 months, 81% when 24 months, and 80% when reached 36 months. These suggested that following to 18 to 24 months, the PFS accessed a platform. According these results, the increase of false negative in our study was approximately at 3% to 5%. It had reflected the short-term effect that could be accepted. Finally, the MRI scan time of this study is too long, such as the time to predict the whole body scan in 1 to 1.5 hours. But gratifying is that we have further optimized scan sequences for scan time compression and are exempt from contrast media injection and waiting time (relative to PET/CT and enhanced CT).

Clinically, it is very important to distinguish between benign lymph nodes and malignant lymphomas between PR after chemotherapy and CR of tumors after chemotherapy. MRI has the potential to assist with these types of clinical decisions.

## 5 Strengths and limitations of this study

- Our study found that diffusion weighted MRI can be used to derive prognostic indicators based on physical attributes of lymphomas and lymph nodes.
- We evaluated the initial stage and efficacy of lymphoma by non-ionizing radiation.
- We screened out high diagnostic indicators using new MR parameters and models.
- This project is an exploratory study with a relatively short follow-up period.

## Acknowledgments

The authors are grateful to Qiang Li, a radiologist, Qiu Li and Zhi Hui Liu, patient representatives at the Affiliated Tumor Hospital of Guangxi Medical University, Nanning 530021, P.R. China.

## Author contributions

Cheng C. Liao and Yun Y. Qin contributed to the conceptualization, visualization, writing of the original draft preparation, review and editing, and data curation. Le Q. Li contributed to the formal analysis and funding acquisition. Hong Cen, Yan Rong Xiao H. Tan, and Qing Ke contributed to the investigation, methodology, project administration, and supervision. Yun Y. Qin, Qi Tang, and Jian H. Zhong contributed to the literature search and proofreading. All authors contributed to the critical revision of this article and read and approved the final version.

**Conceptualization:** Le-Qun Li, Hong Cen.

**Data curation:** Cheng-cheng Liao, Hong Cen.

**Formal analysis:** Cheng-cheng Liao, Qi Tang.

**Funding acquisition:** Le-Qun Li.

**Investigation:** Qi Tang, Le-Qun Li, Hong Cen.

**Methodology:** Cheng-cheng Liao.

**Project administration:** Qi Tang, Jian-Hong Zhong, Le-Qun Li.

**Resources:** Cheng-cheng Liao, Yun-ying Qin.

**Software:** Cheng-cheng Liao, Yun-ying Qin, Qi Tang.

**Supervision:** Cheng-cheng Liao, Xiao-hong Tan, Yan Rong, Hong Cen.

**Validation:** Cheng-cheng Liao, Yun-ying Qin, Xiao-hong Tan, Qing Ke, Hong Cen.

**Visualization:** Cheng-cheng Liao, Yun-ying Qin, Qi Tang, Qing Ke, Yan Rong.

**Writing – original draft:** Cheng-cheng Liao, Qi Tang.

**Writing – review & editing:** Yan Rong, Jian-Hong Zhong, Le-Qun Li, Hong Cen.
